# Bilateral ankle deformities affects gait kinematics in chronic stroke patients

**DOI:** 10.3389/fneur.2023.1078064

**Published:** 2023-02-09

**Authors:** Hogene Kim, Ji-Eun Cho, Kyeong-Jun Seo, Jooyoung Lee

**Affiliations:** ^1^Department of Clinical Rehabilitation Research, National Rehabilitation Center, Seoul, Republic of Korea; ^2^Translational Research Center on Rehabilitation Robots, National Rehabilitation Center, Seoul, Republic of Korea; ^3^Department of Applied Statistics, Chung-Ang University, Seoul, Republic of Korea

**Keywords:** stroke, foot, ankle, 3D scan, anthropometry, deformity, geometric morphometrics, ankle-foot-orthosis

## Abstract

**Objectives:**

Stroke patients suffer from ankle joint deformities due to spastic ankle muscles. This study evaluated the viability of using 3D scanned surface images of the feet of stroke victims to visually assess the deformities of a hemiparetic foot and investigated the influences of deformed ankle joints on gait kinematics.

**Methods:**

A total of 30 subjects with stroke-induced hemiparesis and 11 age-matched healthy controls completed the clinical assessments. We analyzed their feet's morphometric characteristics using a 3D scanner, identified convenient anthropometric measurements, and conducted gait trials on even and uneven terrains. The 3D foot morphometric characteristics were evaluated using the geometric morphometrics method (GMM).

**Results:**

Results showed that there were significant differences in bilateral foot shapes between the chronic stroke patients and healthy controls and between the paretic and non-paretic sides in the chronic stroke patients. In stroke patients, those with the smaller medial malleoli's vertical tilt angles showed significantly different ankle ranges of motion of dorsi-/plantar flexion during gaits on uneven terrains (*p* = 0.009). In addition, those with the greater medial malleoli's vertical tilt angles showed significantly different ankle ranges of motion of inversion/eversion during gaits on even and uneven terrains (*p* < 0.05).

**Conclusion:**

Using 3D scanning technology, bilateral morphometric changes in the feet of chronic stroke patients were shown by GMM and the simple anthropometric measurements identified its shape deformities in the feet. Their possible effects on gait kinematics while walking on uneven terrains were investigated. Current methodology can be potentially useful in applying conventional productions of clinically manufactured, patient-fitted ankle-foot-orthosis in orthotics and prosthetics, and in detecting various unidentified pathological deformities in the feet.

## Introduction

Most patients with upper motor neuron disorders, such as stroke, multiple sclerosis, and amyotrophic lateral sclerosis, have spastic lower limb muscles (1). Specifically, 30–70% of stroke survivors suffer from spastic or stiff ankle muscles (2). Spastic muscles in the ankle joints significantly increase joint stiffness owing to the dependence of the muscle tone on the rising velocity of the stretch reflex (3). Thus, patients with stroke-induced hemiparesis suffer from several movement disorders. Previous studies have reported that the equinus foot condition significantly disrupts the gait patterns of stroke patients (4, 5). Decreased muscle activity in the ankle plantar flexors of such patients reduces the propulsive forces acting during their gait (5). A patient with equinovarus foot requires more time to recover than one without equinus foot and they have to undergo intense rehabilitation (6).

Chronic stroke victims who have not undergone ankle treatments suffer from a constant inequivalence between their major ankle muscles, i.e., the spastic triceps surae and ankle dorsiflexors. This inequivalence is responsible for the geometric deformities of a foot (7). A spastic equinus foot introduces many functional limitations into the daily activities of patients, namely foot drop during gait (4, 8) and balance impairments (9). Various clinical treatments, such as orthopedic surgeries, toxin injections, and ankle-foot-orthoses (AFOs) (10, 11) have been adopted to treat the spastic equinovarus foot condition. AFO is a popular assistive technology that has been used to treat patients having a hemiparetic foot, both inside and outside clinics.

Recent advances in scanned 3D digital imaging technology have been successfully incorporated into clinical practice to enable the treatment of various pathological deformities. For example, standardized measurements of 3D surface images of breasts and head shapes allow for obtaining objective dimensions in a subjective field and overcoming the deficits of conventional 2D photography in plastic and reconstructive surgery. This enables clinicians to reproduce their results and precisely track the deformities of patients (12). Although 3D surface imaging technology can be used in applying it to many patient-fitted assistive devices in rehabilitation clinics, orthotics and prosthetics, the application of 3D surface imaging technology in detecting hemiparetic foot deformities is limited. In addition, the traditional approach to shape analysis, using numerical quantities such as the distance or angle between a foot's landmarks, employs quantities that are less verified than the actual coordinates of the landmarks, ignoring the foot's precise geometry (13).

This study evaluates the viability of using 3D scanned surface images of the feet of stroke patients to visually assess the deformities of a hemiparetic foot. Anthropometric measurements that can locate typical foot landmarks, characteristic of hemiparetic foot abnormalities, are easier to obtain when using modern 3D scanning technology. First, we hypothesized that there was no difference in the foot and ankle shapes (1) between the paretic and non-paretic sides in chronic stroke patients and (2) between the hemiparetic sides of chronic stroke patients and age- and gender-matched healthy controls using the geometric morphometrics method (GMM); variations in foot shapes were examined based on the coordinates of foot landmarks as well as by using the traditional approach. Secondly, we hypothesized that (3) the effect of the anthropometric difference between paretic and non-paretic feet did not affect the gait parameters of chronic stroke patients.

## Methods

### Participants

The subjects were recruited from National Rehabilitation Center (Seoul, South Korea). The eligibility criteria for adults with stroke-induced hemiparesis to participate in this study are listed as follows:

1. The Patient should have experienced foot drop during gait.
2. The ankle dorsiflexor should be moderately strong (manual muscle testing grades are such that 1 corresponds to trace and 4 to good).
3. The patient should have moderate ankle spasticity (modified Ashworth scale ≥2).
4. The patient should understand the contents of the test and follow the instructions (mini-mental status examination ≥24).

The healthy controls included people without mental and physical impairments. The exclusion criteria for the participants are mentioned below:

1. Patients with severe contracture and pain at the ankle joint.
2. Patients unable to walk independently on level ground (Functional Ambulation Categories score <4 and Berg balance scale score <41).
3. Patients with orthopedic complications at the ankle joint.
4. Patients who have attended intensive ankle training sessions within the last 6 months.

The study protocol was reviewed and approved by the local institutional review board (NRC-2019-03-021) and registered at a clinical trial registry in the World Health Organization registry network (Clinical Research Information Service, KCT0005171). The methodology of the study was explained in depth to all the participants. Written informed consent was obtained from each participant prior to data collection.

### Foot anthropometric measurements

To quantify foot shape, landmark-based geometric morphometrics were used (14). In accordance with commonly used foot anatomical landmarks, a set of four 3D landmarks on the foot surface were chosen that effectively show the hemiparetic deformity in foot and ankle structure of chronic stroke patients: (1) the first metatarsal joint head, (2) the fifth metatarsal joint head, (3) medial malleolus, and (4) lateral malleolus (15–18). Then we have set three points of a foot as custom references to define the plane of the foot ground-contact-area while standing: (5) the medial and (6) the lateral outmost point of the heel ground-contact-area while standing, (7) the tip of index toe, and the origin as the most posterior point of the foot along the line between the calcaneus center and index toe on this plane. All these landmarks were recorded on the paretic and non-paretic sides for each stroke patient and on the dominant side for each healthy control. We reflected on the non-paretic or non-dominant side of the foot for landmark correspondence. The original landmarks (right side) and the ones we reflected on (left side) were then superimposed using Generalized Procrustes Analysis (GPA) to remove the effects of position, size, and orientation (19). Procrustes coordinates were then used to analyse the foot shape variations between the stroke patients and healthy controls and between the paretic and non-paretic asymmetry in foot shapes for stroke patients.

**Figure 1:**
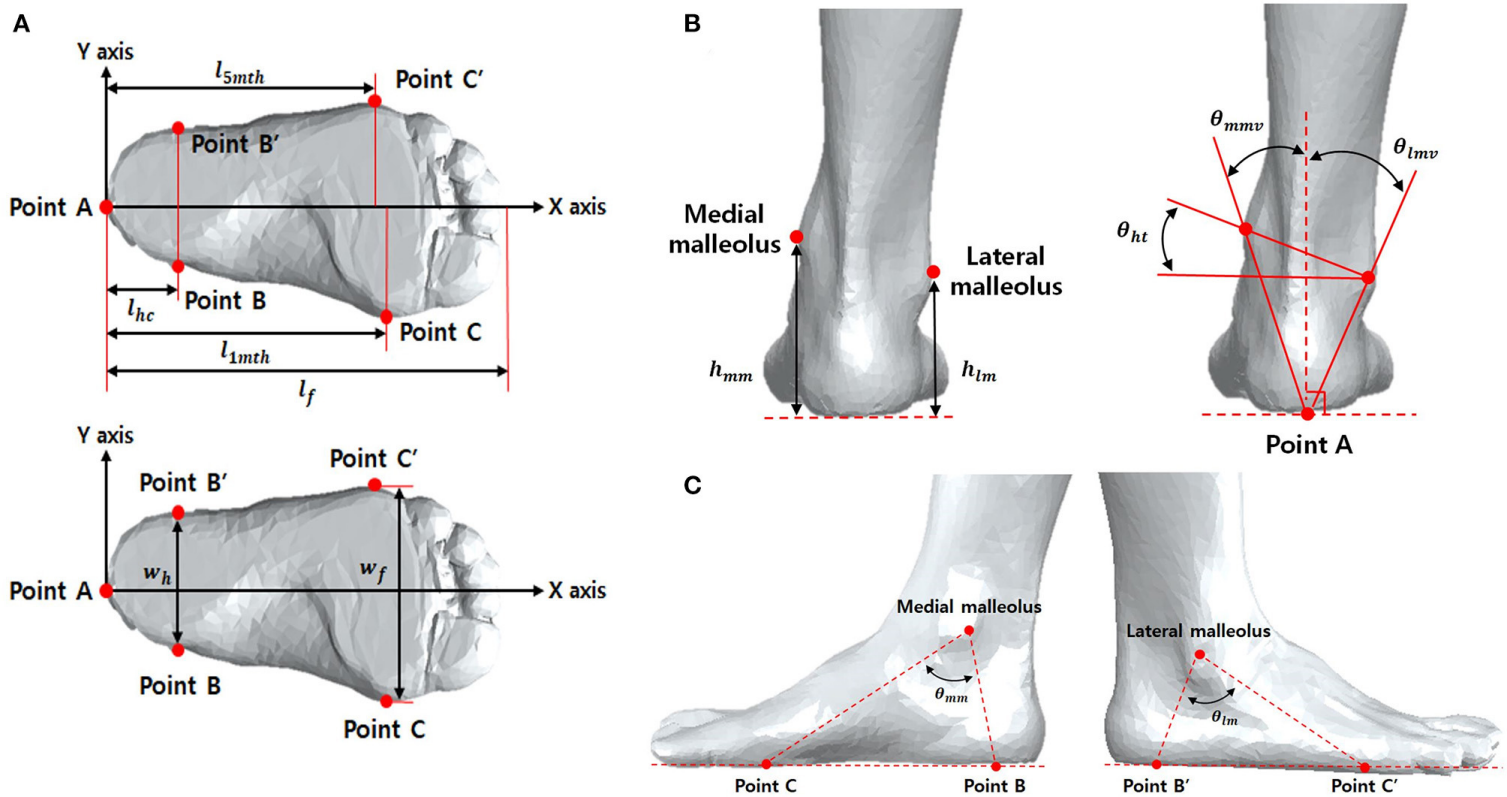
Reference points for 3D ankle measurements in transverse plane **(A)** and frontal plane **(B)**, and sagittal plane **(C)**. *l*_*foot*_, length of foot; *l*_1*mt*_, length of 1st metatarsal head; *l*_5*mt*_, length of 5th metatarsal head; *l*_*hc*_, length of hind foot center; *w*_*h*_, width of hind foot; *w*_*f*_, width of forward foot; *h*_*mm*_, height of medial malleolus; *h*_*lm*_, height of lateral malleolus; θ_*mmv*_, vertical degree of medial malleolus; θ_*lmv*_, vertical degree of lateral malleolus; θ_*ht*_, degree of horizontal tilt; θ_*mm*_, degree of medial malleolus; θ_*lm*_, degree of lateral malleolus; θ_*mv*_, vertical degree of malleolus.

In addition, the traditional approach to shape analysis including using distances and angles between landmarks, was applied. The angles θ_ht_, θ_mmv_, θ_lmv_, θ_mm_, and θ_lm_ represent the horizontal tilt, medial malleolus' vertical tilt, lateral malleolus' vertical tilt, medial malleolus' side, and lateral malleolus' side, respectively. Here, θ_ht_ denotes the angle between the horizontal line in the coronal plane and the line connecting the medial and lateral malleoli, θ_mmv_ denotes the angle between the vertical line in the coronal plane and the line connecting the origin to the medical maliolus, θ_lmv_ does to lateral malleolus θ_mm_ denotes the angle between the lines connecting from (1) and (5) to the medial malleolus, and θ_lm_ does connecting from (2) and (6) to the lateral malleolus. These parameters are shown in Figure 1. All foot lengths, widths, and heights were normalized according to the individual's full length of the foot (l_f_) and expressed as a percentage.

### Clinical assessments

The following standard clinical measurements of the subjects were obtained prior to the procedure:

1. Ankle isometric contraction force (*N*);

To measure ankle strength, the maximal voluntary isometric contraction force of the paretic ankle muscle was measured using a portable manual muscle strength tester (Lafayette Instrument, Lafayette, IN, USA). The participants were in the sitting position during the measurements, and resistance was provided using a measurer to isolate the ankle joint motion. Each trial consisted of a voluntary isometric contraction with a maximum of 3 s; a 30 s rest period was provided between trials. The average value was used for analysis.

2. Modified Ashworth scale (MAS, score);

To testing the spasticity of plantar flexor, place the ankle joint a maximally plantarflexion position and move to a position of maximal dorsiflexion over 1 s. A five-point ordinal scale described the grades of resistance encountered during such passive muscle stretching.

3. Berg balance scale (BBS, score);

To measure balance, the BBS was used as a clinical test of an individual's static and dynamic balance. The test comprised a set of 14 simple balance-related tasks ranging from standing from a sitting position to standing on one foot.

4. Fugl-Meyer assessment of-Lower Extremity (FMA-LE, score);

To measure the motor function, we used the motor domain of the FMA-LE. The motor domain includes measurements of movement, coordination, and reflex action of the hip, knee, and ankle. The maximum possible score of the motor domain of the FMA-LE is 34, corresponding to full sensorimotor recovery.

5. Fall efficacy scale (FES, score);

The FES was applied to ascertain an individual's level of confidence when performing activities of daily living. It is a self-report questionnaire that contains 10 items, with each scored using a scale of 0 to 10, and the total summed scores range from 0 to 100. A high score indicates high confidence when performing activities of daily living without falling.

### Foot assessments

Each subject placed their target foot comfortably on a custom, transparent footplate that was tilted by 45° and was supported 50 cm above the ground by a central pillar. This arrangement ensured that the subject's ankle joint remained in a neutral position while they were in a semi-sitting posture on a comfortable chair with their knee in a naturally flexed position (see Supplementary Figure 1). The orientation of the ankle joint was neutral, and moderate pressure was applied to the surface of the footplate. A test administrator then acquired paretic and non-paretic 3D images of the foot and ankle structures using a commercial 3D scanner (Artec Eva, Artec 3D Inc., Luxembourg), weighing 0.9 kg with a resolution of 0.5 mm. The acquired 3D images were stored in the STereoLithography (STL) file format and post-processed using a 3D CAD program (SolidWorks, Dassault Systems, Waltham, MA, United States). From the processed images, the 3D foot anthropometric characteristics were identified, and the geometric parameters of the foot were measured.

### Gait assessments

Each subject walked three times on 10 m even and uneven terrains at a self-selected comfortable speed, as presented in a previous study. The uneven terrain was created using randomly arranged triangular wooden prisms (H 1.5 cm × W 3.5 cm × L 6–12 cm) placed under a 1.5 m × 10 m strip of industrial carpeting with a surface texture identical to that of the even surface (20). Kinematic data were recorded at 100 Hz with 24 reflective passive markers on the lower limbs using a 3D motion capturing system based on a commonly used model (Plug-in-Gait, Vicon, Oxford, UK). The joint and gait parameters data were then processed using Visual3D (C-Motion, Germantown, MD, USA) to analyse joint kinematics of the hip, knee, ankle, and gait parameters during gait on even and uneven terrains.

### Statistical analysis

A statistical shape analysis was performed using the R package geomoph v.4.0.0 (21). Before all analysis, normality of data distribution was tested. A Procrustes Analysis of variance (ANOVA) on Procrustes distances was conducted to test the significant foot shape differences between the stroke patients and healthy controls. The patterns of overall shape variation were visualized using Principal Component Analysis (PCA). The shape analysis of bilateral symmetry was performed using a Procrustes ANOVA with individual and side (as the paretic and non-paretic foot of each stroke patient) and individual^*^side interaction. Symmetric variations among the individuals corresponded to the main effect of the individual. Asymmetry, expressed as the difference between the paretic and non-paretic sides of each stroke patient, was partitioned into two components: (i) the directional asymmetry (DA) as the difference between the average of the paretic and non-paretic sides, and (ii) fluctuating asymmetry (FA) as the individual variability of paretic/non-paretic differences. In ANOVA, DA corresponded to the main effect of the side and FA corresponded to the individual^*^side interaction. PCA was also performed to visualize the patterns of fluctuating asymmetry variations. Permutation tests with 10,000 iterations were conducted for testing the ANOVA effects with a significance level of 0.05.

The statistical comparisons between the intra- and inter- variables were performed using the Statistical Package for the Social Sciences (SPSS Inc., Chicago, Illinois; version 12.0). Before all analyses, the normality of data was assessed using the one-sample Kolmogorov-Smirnov test. The Mann-Whiteny U-test was performed on the variables for a between-group comparison, i.e., strokes vs. healthy controls and upper vs. lower halves of foot deformity, while the Wilcoxon signed rank test was performed for a within-group comparison, i.e., paretic vs. non-paretic patients. Analysis of covariance (ANCOVA) was performed for a comparison between groups by controlling weight and gender as covariates. We applied Bonferroni correction for 30 tests to correct for multiple comparison. The effects of foot deformities on step and joint kinematics during gait were investigated based on the results of the Wilcoxon signed-rank test. These results were used to compare the walking kinematics of the upper and lower halves of the participants for each kinematic deformity of their feet. The alpha level was set to P < 0.05 to identify the statistically significant readings. The appropriate number of subjects for the study was estimated by performing a power analysis to calculate the values of alpha and beta.

## Results

### Participant characteristics

A total of 30 subjects with stroke-induced hemiparesis and 11 age-matched healthy controls participated in this study (Supplementary Table 1). The stroke patients were all independent walkers and completed the clinical assessments. The weights of the stroke patients were significantly higher than those of the healthy controls (*p* = 0.005). The ankle joint strengths of the stroke patients were significantly lower than those of the healthy controls (*p* < 0.001).

### Foot and ankle morphometrics and anthropometry

The length, height, and width were normalized with respect to the total foot length and expressed in terms of percentages (%) (Table 1). There was a significant difference between the length of the 1st metatarsal head, width of the forward foot, and vertical tilt degrees of the medial and lateral malleoli of the paretic and non-paretic sides for the stroke patients (*p* < 0.05). The ANCOVA analysis showed a significant difference for θ_mm_ (*p* = 0.002) using weights as a covariate and θ_mm_ (*p* = 0.001) and θ_lm_ (*p* = 0.001) using gender as a covariate. After applying Bonferroni correction W_h_ and θ_mm_ were no longer significant.

**Table 1:** 3D ankle anthropometric measurements (*N* = 41).

		**Stroke (*n =* 30)**		**Healthy (*n =* 11)**	***p-*** **values**				
		**Paretic**	**Non-paretic**	**Dominant**	**P-NP**	**P-D**	**P-D** ^⋆^	**P-D** ^⋆⋆^	**NP-D**
Length (%)	*l*_1mt_	73.9 ± 2.2	71.1 ± 1.9	73.2 ± 0.2.1	**<0.001** ^*^	0.222	0.175	0.470	**0.002** ^*^
	*l*_5mt_	64.0 ± 2.3	64.0 ± 2.0	63.6 ± 2.5	0.888	0.556	0.625	0.769	0.680
	*l*_hc_	19.0 ± 1.8	19.7 ± 1.8	17.9 ± 1.6	0.099	0.102	-	-	**0.019** ^*^
Width (%)	*w*_h_	28.0 ± 2.3	28.0 ± 2.1	26.5 ± 2.2	0.988	0.137	0.316	0.122	0.075
	*w*_f_	39.7 ± 1.7	40.9 ± 2.4	40.4 ± 2.4	**0.049** ^*^	0.361	0.482	0.442	0.556
Height (%)	*h*_mm_	38.7 ± 2.7	39.9 ± 3.0	36.4 ± 2.5	0.133	**0.029** ^*^	0.116	0.061	**0.002** ^*^
	*h*_lm_	30.0 ± 2.1	30.5 ± 1.7	29.1 ± 2.2	0.206	0.385	0.519	0.490	0.052
Angle (deg)	θ_mmv_	20.5 ± 4.8	18.4 ± 4.3	22.7 ± 2.1	0.037^*^	**0.049** ^*^	0.268	0.324	**0.001** ^*^
	θ_lmv_	29.1 ± 3.7	30.1 ± 3.9	28.5 ± 2.4	0.117	0.988	-	-	0.068
	θ_ht_	15.9 ± 5.6	15.9 ± 3.6	13.8 ± 1.2	0.379	0.517	-	-	**0.029** ^*^
	θ_mm_	55.8 ± 3.5	53.8 ± 4.0	60.7 ± 4.4	**0.026** ^*^	**0.002** ^*^	**0.002** ^*^	**0.001**	**<0.001** ^*^
	θ_lm_	60.1 ± 3.8	56.1 ± 2.8	66.0 ± 6.1	**<0.001** ^*^	**0.005** ^*^	-	**0.001**	**<0.001** ^*^

Comparisons made by Mann Whitney U tests, Wilcoxon signed rank tests, ANCOVA analysis ⋆ using weights as covariates, and ANCOVA analysis ⋆⋆ using gender as covariates.

P-NP, Paretic-Non paretic; P-D, Paretic-Dominant; NP-D, Non paretic-Dominant; *l*_1*mt*_, length of 1st metatarsal head; *l*_5*mt*_, length of 5th metatarsal head; *l*_*hc*_, length of hind foot center; *w*_*h*_, width of hind foot; *w*_*f*_, width of forward foot; *h*_*mm*_, height of medial malleolus; *h*_*lm*_, height of lateral malleolus; θ_*mmv*_, vertical degree of medial malleolus; θ_*lmv*_, vertical degree of lateral malleolus; θ_*ht*_, degree of horizontal tilt; θ_*mm*_, degree of medial malleolus; θ_*lm*_, degree of lateral malleolus.

* p < 0.05.

The GMM showed that the foot shapes of stroke patients significantly differed in the paretic side (*F* = 3.298, *p* = 0.001) and the non-paretic side (*F* = 2.184, *p* = 0.034) compared to the dominant side in healthy controls. The foot shape group differences were observed along the first two principal components (PCs) primarily in the medial malleolus, resulting in a smaller degree of change in the medial malleolus for the paretic and non-paretic sides of stroke patients than the healthy controls (Supplementary Figure 2). The Procrustes ANOVA done for testing bilateral symmetry showed no significant variations among individuals (symmetry: SS = 0.190, d*f* = 29, *F* = 1.147, *p* = 0.123) and no significant foot shape variations between the paretic and non-paretic sides (directional asymmetry: SS = 0.009, d*f* = 1, *F* = 1.622, *p* = 0.116), The shape directional asymmetry showed a subtle foot shape difference in the first metatarsal joint head, the medial malleolus, and the index toe. The first principal component (PC1) explained that 31.8% of fluctuating asymmetry was related to overall locations. The principal component (PC2) explained that 15.2% was primarily related to the medial and lateral malleoli and the first metatarsal joint head, indicating that a foot with a positive PC2 was tilted medially.

### Kinematics during gait

There were significant differences between the joint kinematics and gait parameters of the stroke patients and healthy controls (Table 2). All hip, knee, and ankle range of motion (ROM) except only hip abduction-adduction of the paretic side were significantly less than that of the non-paretic side while walking on both even and uneven terrains (*p* < 0.01). In addition, the step length and step time of the paretic side during gait on even terrains and step time of the paretic side during gait on uneven terrains were significantly less than those of the non-paretic side (*p* < 0.01). All joint kinematics except only ankle inversion-eversion of the paretic side of stroke patients were significantly less than that of the dominant side of healthy control while walking on both even and uneven terrains (*p* < 0.01). Furthermore, there were significant difference in all gait parameters except only stance time between the paretic side of stroke patients and the dominant side of healthy control while walking on both even and uneven terrains (*p* < 0.01).

**Table 2:** Joint kinematics and gait parameters on even and uneven terrains between strokes and healthy controls.

	**Surface Type**	**Joint movements**	**Stroke (*n =* 30)**		**Healthy (*n =* 11)**	***p-*** **value**	
			**Paretic**	**Non-paretic**	**Dominant**	**P-NP**	**P-D**
**Joint kinematics** (deg)	Even	Ankle DF-PF	**14.8** **±7.6**	**20.6** **±8.6**	**30.0** **±4.2**	<0.001^*^	<0.001^*^
		Ankle IV-EV	**6.5** **±4.6**	**8.6** **±5.9**	6.7 ± 1.9	0.002^*^	0.837
		Hip Flex-Ext	**26.3** **±14.1**	**37.1** **±16.2**	**49.2** **±5.3**	<0.001^*^	<0.001^*^
		Hip Abd-Add	**8.5** **±3.8**	9.9 ± 4.0	**15.1** **±2.1**	0.091	<0.001^*^
		Knee Flex-Ext	**34.7** **±19.4**	**50.5** **±20.3**	**68.3** **±5.4**	<0.001^*^	<0.001^*^
	Uneven	Ankle DF-PF	**20.8** **±6.0**	**27.5** **±5.0**	**28.2** **±4.4**	<0.001^*^	0.002^*^
		Ankle IV-EV	**8.4** **±5.5**	**9.4** **±4.2**	6.6 ± 2.1	0.013^*^	0.150
		Hip Flex-Ext	**32.7** **±11.2**	**45.8** **±7.5**	**53.0** **±5.1**	<0.001^*^	<0.001^*^
		Hip Abd-Add	**10.5** **±3.7**	11.6 ± 3.0	**14.9** **±2.3**	0.252	0.002^*^
		Knee Flex-Ext	**45.0** **±16.7**	**61.1** **±7.3**	**68.3** **±5.9**	<0.001^*^	<0.001^*^
**Gait parameters**	Even	Walking speed (cm/sec)	**52.4 ± 0.2**		**112.2** **±17.9**	–	<0.001^*^
		Step length (cm)	**28.2** **±8.6**	**32.3** **±8.2**	**51.2** **±6.0**	0.025^*^	<0.001^*^
		Step width (cm)	**14.8** **±4.0**	14.4 ± 5.0	**7.0** **±2.9**	0.526	<0.001^*^
		Step time (sec)	**0.9** **±0.2**	**1.0 ± 0.2**	**0.7** **±0.1**	<0.001^*^	<0.001^*^
		Stance time (sec)	0.4 ± 0.1	0.4 ± 0.1	0.4 ± 0.0	0.391	0.085
		Swing time (sec)	**0.9** **±0.2**	0.9 ± 0.2	**0.7** **±0.1**	0.405	<0.001^*^
		Percentage of stance time (%)	**0.3** **±0.0**	0.3 ± 0.0	**0.4** **±0.0**	0.491	0.002^*^
	Uneven	Walking speed (cm/sec)	**36.3** **±23.8**		**107.4** **±16.9**	–	<0.001^*^
		Step length (cm)	**21.6** **±12.0**	23.9 ± 13.3	**51.3** **±4.3**	0.127	<0.001^*^
		Step width (cm)	**15.7** **±5.0**	16.2 ± 4.2	**8.3** **±3.4**	0.814	<0.001^*^
		Step time (sec)	**0.9** **±0.2**	**1.0 ± 0.3**	**0.7** **±0.1**	0.007^*^	0.003^*^
		Stance time (sec)	0.4 ± 0.1	0.4 ± 0.1	0.4 ± 0.0	0.381	0.898
		Swing time (sec)	**1.0** **±0.2**	1.0 ± 0.3	**0.8** **±0.1**	0.666	0.003^*^
		Percentage of stance time (%)	**0.3** **±0.1**	0.3 ± 0.1	**0.4** **±0.0**	0.518	0.012^*^

Comparisons made by Mann Whitney U tests and Wilcoxon signed rank tests.

P-NP, paretic-non paretic; P-D, paretic-dominant; DF-PF, dorsiflexion-plantarflexion; IV-EV, inversion-eversion; Flex-Ext, flexion-extension; Abd-Add, abduction-adduction.

* p < 0.05.

### Foot anthropometry and gait kinematics

**Figure 2:**
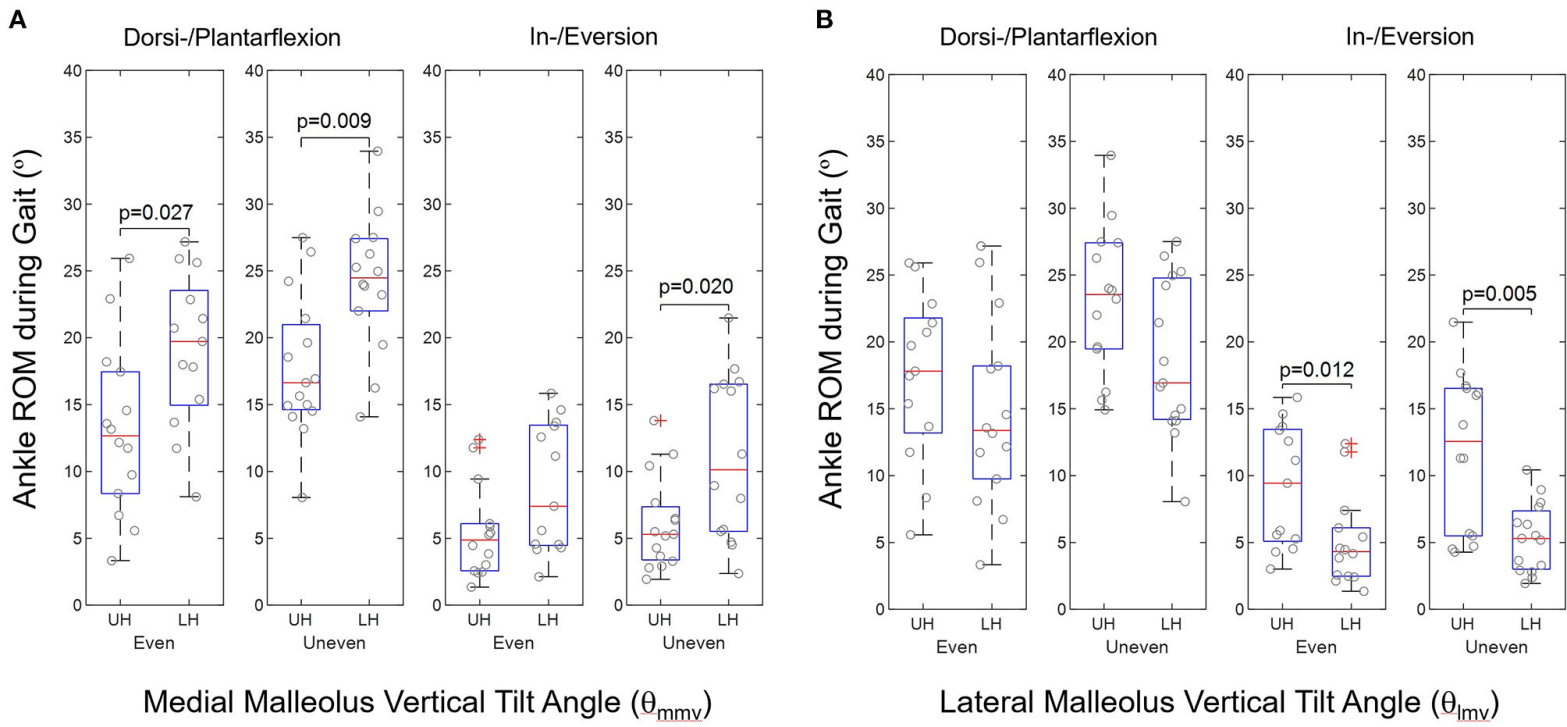
Group comparison of ankle ROM during walking in terms of upper half (UH) and lower half (LH) in **(A)** medial malleolus vertical tilt angle (θ_mmv_) and **(B)** lateral malleolus vertical tilt angle (θ_lmv_) according to ankle geometric measurements in stroke (*n* = 30).

There were significant differences between the ankle joint kinematics of the upper and lower halves of the medial and lateral melleoli's vertical tilt angles, θ_mmv_ and θ_lmv_, respectively, during gait on the even and uneven terrains, as shown in Figure 2. The lower half of the medial malleolus' vertical tilt angle (θ_mmv_), i.e., smaller θ_mmv_, had significantly greater ankle ROM than those of upper half in the sagittal and frontal planes during gait on uneven terrains (dorsi-/plantarflexion: *p* = 0.009, in-/eversion: *p* = 0.020, Figure 2A). The upper half of the lateral malleolus' vertical tilt angle (θ_lmv_), i.e., greater θ_lmv_, had significantly greater ankle ROM in the frontal planes during gait on both even and uneven terrains (even: *p* = 0.012, uneven: *p* = 0.005, Figure 2B).

## Discussion

Our study successfully showed that using 3D scanning technology, the foot morphometric changes in the feet if chronic stroke patients and their anthropometric measurements identified significant hemiparetic deformities in ankle and foot structures between the paretic and non-paretic sides as well as between the chronic stroke patients and healthy controls. Simple measurements using typical foot landmarks identified hemiparetic ankle deformities. Then there were significant differences in the joint and gait kinematics between chronic stroke patients and healthy controls when they walked on even and uneven terrains, with respect to malleoli measurements.

The results in this study have a couple of clinical and biomechanical implications. First, our results revealed that chronic stroke patients had significant shape deformities in the paretic and non-paretic sides, compared to healthy controls. The chronic stroke patients who participated in this study had an average 10 years onset stroke period. These people usually had pathological conditions in ankle muscles such as spasticity in the triceps surae, proprioceptive deficits, and weaknesses in ankle dorsiflexor muscles (22, 23). Ankle biomechanics, which affected by these pathological conditions, consists of a complex form involving subtalar joints rather than simple flexion-extension movements at talocrural joints. A previous study on ankle stability and morphological factors revealed that the bone morphology of the talus showed potential instability in plantar flexion and the radii of curvature of the talar dome have a variable mediolateral distribution (24). In addition, ligament components such as cervical and anterior talofibular ligaments and talocalcaneal ligaments are largely responsible for inversion, the most advanced injury mechanism (24). Compared to previous studies based on CT measurements, this study was able to compare ankle morphological changes in stroke patients with a relatively simple 3D surface image. Even if it does not include fundamental morphologies such as bone and ligament, 3D surface images can be a way to measure pathological morphological changes. Moreover, foot geometry and ankle stiffness are two crucial design aspects for an AFO. Therefore, the 3D morphometric changes in a post-stroke hemiparetic foot must be considered while designing AFOs.

Second, there was a significant difference in ankle ROM during gait with respect to ankle anthropometric differences within stroke patients (especially medial/lateral malleolus vertical tilt angle). Although there are various internal variables that contribute to walking in stroke patients, balance represents one of the largest contributing factors to gait dysfunctions (25). Hence, this study recruited subjects who could walk independently and had good balance (Functional Ambulation Categories score: 4.7 ± 0.5 and BBS score: 47.6 ± 5.3) in order to exclude the effect of poor balance on gait ability as much as possible. The results of this study reveal that foot deformity can contribute to one of the causes of ankle-foot kinematics during even and uneven gait in stroke patients. Spasticity in the ankle and foot muscle groups is very common and often results in various ankle and foot deformities and spastic equinovarus foot is the most common (23, 26, 27). These pathological conditions in stroke patients resulted in the consequential inequivalences of muscle tone and force and reduced ankle joint ROM during gait. Joint damage, deformity, and pain in this population also cause abnormal joint loading, inefficient energy expenditure, and falls during walking (28). In addition, limited ROM in ankle-foot joints cause the compensatory movements of proximal joints (29). A previous study demonstrated that there is a large difference in kinematics at ankle joint between the stroke patients and healthy control in uneven terrains and the subtalar joint ROM in stroke patients is considerably reduced compared with healthy control (30). Therefore, practically measured in clinics, the identified 3D foot anthropometric quantities, such as the medial malleolus height (*h*_mm_) or tilt angle (*q*_mmv_), may be effectively and conveniently useful in assessing the ankle deformity that would potentially relate ankle joint kinematics during gait on even and uneven terrains. Future studies can analyse the relationship between the morphometric changes of ankles and their mobility during gait under different environmental factors. These factors could include various terrain conditions, such as slippery, irregular, or inclined surfaces. The stroke patients were found to have shorter but wider steps than those of the healthy controls in this study, indicating a reduced gait efficiency in the former, corresponding to reduced energy. Our future study would focus on the impact of ankle shape deformity on related factors such as gait kinematics, balance capability, metabolic cost, etc.

Third, the 3D scanned ankle surface images of stroke patients would have clinical implications to follow up long-term anthropometric changes of hemiparetic feet for clinical analyses, evaluations, and managements. In the clinical practices, the proposed ankle measurements may be useful for chronic stroke patients as an initial assessment of walking capability during activities of daily livings. The clinic-based conventional assessments on gait kinematics generally involve comprehensive optical-technology equipment and post-processing efforts. Nevertheless, the proposed method may be advantageous in clinical aspects such as less expensive and time consuming, rather used as a simple initial screening one, even done at home. As the height and weight were used as criterion over child's developments, the proposed ankle deformity measurement would potentially contribute to a criterion-referenced tests for patients with neurological disorders, with the predetermined standard. In addition, stroke survivors use a series of AFOs after the occurrence of stroke to prevent foot drop while walking, which may lead to a fatal fall or other injuries (31). Recently, AFO production using 3D scanned ankle surface images has seen several improvements, compared to conventional handmade AFO made from plaster, in terms of production time, cost, design aesthetics, patient-fitted comfort and biomechanical advantages (32). Reductions in size and cost and simultaneous improvements in the accuracy and precision of modern 3D handheld scanners enable rehabilitation clinicians to conveniently visualize 3D surface images. This facilitates better diagnostic analyses and makes using these scanners in therapeutic procedures easier.

## Limitations

This study has several limitations. First, the current analysis does not include an analysis on the underlying bone shape or ligament factors, which directly affect foot shape. This study was unable to show the internal biomechanical aspects of hemiparetic foot shape changes including ankle spasticity, muscle weakness, and ankle stiffness. Second, our 3D foot morphometric data were cross-sectional data in which we assessed static group differences in foot shapes. Further studies using dynamic foot morphometric data in the prospective study will enable us to examine dynamic foot deformities. Third, limited subject population may not reveal more specific shape discrimination between paretic and non-paretic foot shapes, including anthropometric parameters on lateral malleolus. Fourth, this study included only dominant side of healthy control. Fifth, the number of patients and healthy groups was not the same. Sixth, applications of current methods, although suggesting simple anthropometric foot measurements, should follow more thorough evaluations for the clinical validity in the future.

## Conclusion

This study successfully utilized anthropometric parameters that were identified from the 3D scanned surface images of the feet of stroke survivors to distinguish between hemiparetic feet with deformities and healthy feet. The morphometric changes in the hemiparetic feet were used to identify gait changes in stroke patients. The proposed ankle measurements may be useful as an initial assessment of walking capability for chronic stroke patients.

## Data availability statement

The original contributions presented in the study are included in the article/Supplementary material, further inquiries can be directed to the corresponding author.

## Ethics statement

The study protocol was reviewed and approved by the Local Institutional Review Board (NRC-2019-03-021) and registered at a clinical trial registry in the World Health Organization registry network (Clinical Research Information Service, KCT0005171). The patients/participants provided their written informed consent to participate in this study.

## Author contributions

Conceptualization, writing—original draft preparation, writing—review and editing, supervision, and funding acquisition: HK. Methodology: HK and JL. Validation: K-JS, JL, and HK. Formal analysis: J-EC, JL, and K-JS. Visualization: J-EC, JL, and HK. Project administration: K-JS. All authors have read and agreed to the publisheds version of the manuscript.

## Supplementary material

Supplementary Figure 1 Analysis of Geometric Morphometrics on foot and ankle shapes in participants. **(A)** paretic foot vs healthy foot, **(B)** non-paretic foot vs healthy foot, and **(C)** paretic vs non-paretic sides. ^*^a1 and b1 indicate Scatterplots of the first two principal components of the foot and ankle shape variations between stroke patients and healthy controls. (a2 and a3) and (b2 and b3) indicate patterns of shape variation in stroke patients and healthy controls - paretic side (a2 and a3) and non-paretic side (b2 and b3). The red line represents maximum (positive) values, and the yellow line represents minimum (negative) values along PC1 and PC2 (the bottom panel). c1 indicate the directional asymmetry defined as the difference between the average of paretic and non-paretic sides and (b1 and b2) indicate patterns of fluctuating asymmetry. The red line represents maximum (positive) values, and the yellow line represents minimum (negative) values along PC1 and PC2.

Supplementary Figure 2 Subject's Foot placement during 3D scanning measurements.

Supplementary Table 1 Participants characteristics (*N* = 41).
