# Supplementary figures and images for: Bilateral ankle deformities affects gait kinematics in chronic stroke patients
Source: Front Neurol. 2023 Feb 9;14:1078064. doi: 10.3389/fneur.2023.1078064 (PMC9947404; doi:10.3389/fneur.2023.1078064)

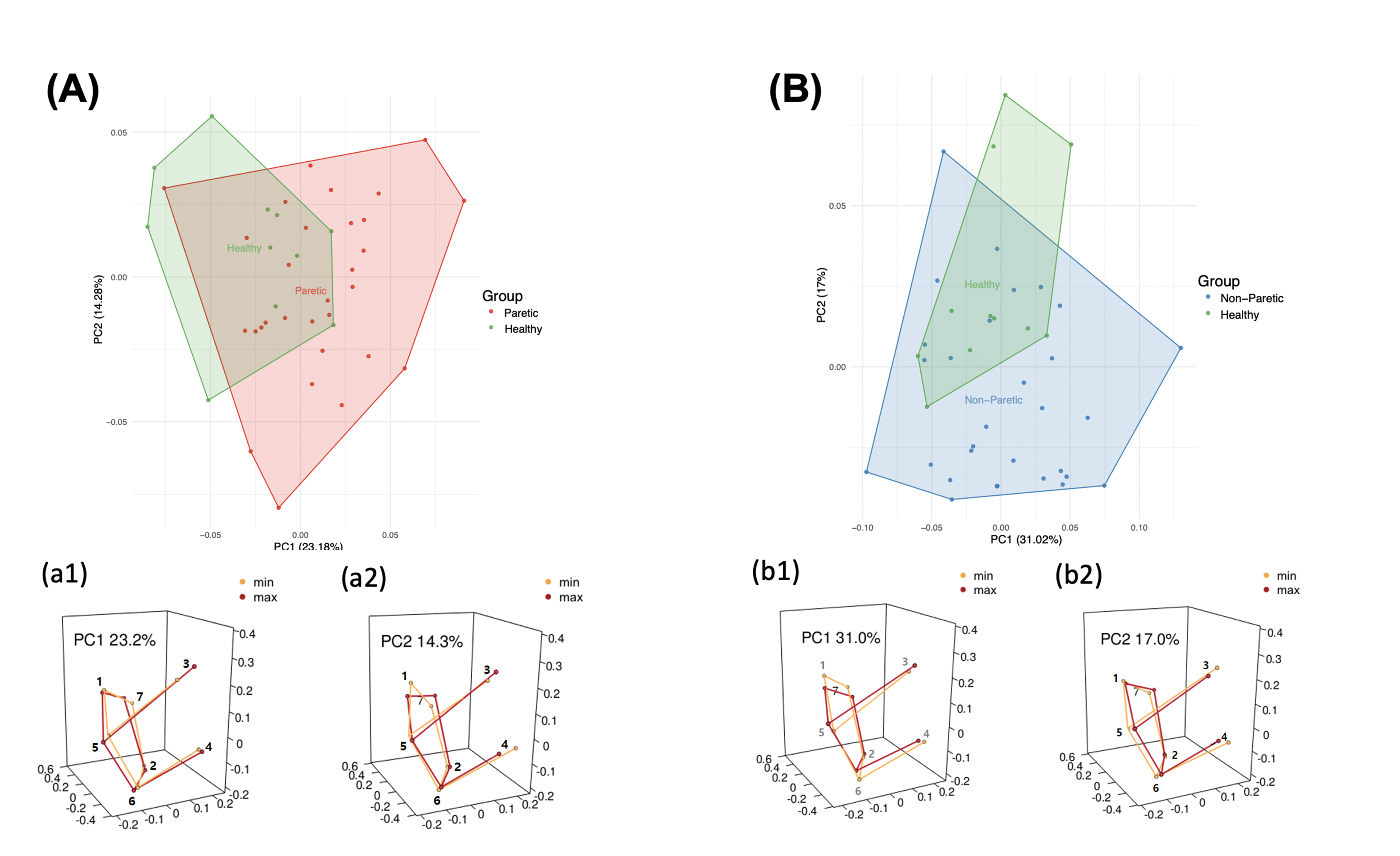

Supplement: Supplementary Figure 1 — Analysis of Geometric Morphometrics on foot and ankle shapes in participants. (A) paretic foot vs healthy foot, (B) non-paretic foot vs healthy foot, and (C) paretic vs non-paretic sides. *a1 and b1 indicate Scatterplots of the first two principal components of the foot and ankle shape variations between stroke patients and healthy controls. (a2 and a3) and (b2 and b3) indicate patterns of shape variation in stroke patients and healthy controls - paretic side (a2 and a3) and non-paretic side (b2 and b3). The red line represents maximum (positive) values, and the yellow line represents minimum (negative) values along PC1 and PC2 (the bottom panel). c1 indicate the directional asymmetry defined as the difference between the average of paretic and non-paretic sides and (b1 and b2) indicate patterns of fluctuating asymmetry. The red line represents maximum (positive) values, and the yellow line represents minimum (negative) values along PC1 and PC2. [file Image_1.TIF]

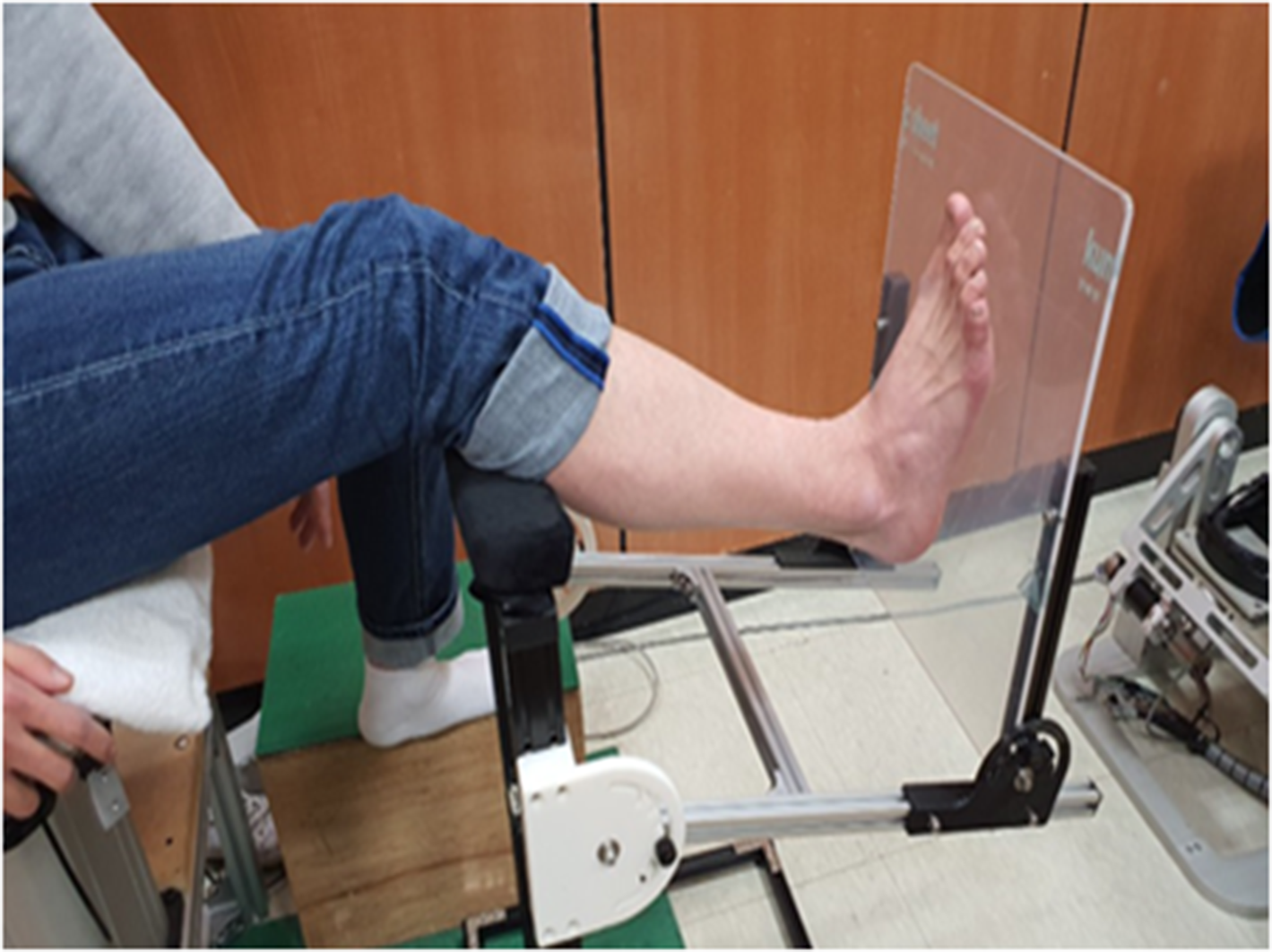

Supplement: Supplementary Figure 2 — Subject's Foot placement during 3D scanning measurements. [file Image_2.PNG]
