# Supplementary material for: Bilateral ankle deformities affects gait kinematics in chronic stroke patients
Source: Front Neurol. 2023 Feb 9;14:1078064. doi: 10.3389/fneur.2023.1078064 (PMC9947404; doi:10.3389/fneur.2023.1078064)
Supplement: Supplementary Table 1 — Participants characteristics (N = 41). [file Table_1.docx]

**Supplemental Table 1** Participants characteristics (N=41)

| Characteristic | | Stroke (n=30) | Healthy (n=11) | *p-*value |
| --- | --- | --- | --- | --- |
| Age (y) | | 65.6±8.7 | 62.9±5.6 | 0.344 |
| Sex, male n (%) | | 19 (63) | 4 (36) | 0.129 |
| Height (cm) | | 165.0±9.8 | 161.5±7.9 | 0.305 |
| Weight (kg) | | **67.2±8.5** | **58.1±8.4** | **0.005*** |
| Dominant side, right n (%) | | 30 (100) | 10 (90) | 0.099 |
| Years since onset | | 10.2±8.7 | - | - |
| Paretic side, right n (%) | | 18 (60) | - | - |
| Functional ambulation categories (score) | | 4.7±0.5 | - | - |
| Berg balance scale (score) | | 47.6±5.3 | - | - |
| Fugl-Meyer lower extremity (score) | | 21.4±5.2 | - | - |
| Length of foot (mm) | | 228.6±16.1 | 227.1±13.2 | 0.776 |
| Ankle MAS (0/1/1+) | | 6/17/7 | - | - |
| Ankle Strength (N) | Dorsiflexion | **8.3±4.2** | **20** | **<0.001*** |
|  | Plantarflexion | **10.9±4.3** | **20** | **<0.001*** |
|  | Inversion | **6.4±2.8** | **20** | **<0.001*** |
|  | Eversion | **5.7±2.3** | **20** | **<0.001*** |

NOTE. Comparisons made using Independent t-tests.

**p* < 0.05.
